# Effect of Comprehensive Interventions Including Nutrition Education and Physical Activity on High Blood Pressure among Children: Evidence from School-Based Cluster Randomized Control Trial in China

**DOI:** 10.3390/ijerph17238944

**Published:** 2020-12-01

**Authors:** Haiquan Xu, Yanping Li, Xianwen Shang, Songming Du, Qian Zhang, Ailing Liu, Guansheng Ma

**Affiliations:** 1 Institute of Food and Nutrition Development, Ministry of Agriculture and Rural Affairs, Beijing 100081, China; xuhaiquan@caas.cn; 2 Department of Nutrition, Harvard T. H. Chan School of Public Health, Boston, MA 02115, USA; yanping@hsph.harvard.edu; 3 School of Behavioural and Health Sciences, Australian Catholic University, East Melbourne, VIC 3002, Australia; xianwen.shang@unimelb.edu.au; 4 Chinese Nutrition Society, Beijing 100022, China; dusm9709@126.com; 5 National Institute for Nutrition and Health, Chinese Centre for Disease Control and Prevention, Beijing 100050, China; zhangqian7208@163.com (Q.Z.); liuailing72@126.com (A.L.); 6 Department of Nutrition and Food Hygiene, School of Public Health, Peking University, Beijing 100191, China

**Keywords:** children, obesity, comprehensive intervention, nutrition education, physical activity, blood pressure, high blood pressure, hypertension

## Abstract

Childhood hypertension has increasingly become a public health problem globally. However, limited literature research examined the effect of comprehensive interventions including nutrition education and physical activity on blood pressure among children. A total of 6764 children aged 7–13 years were analyzed based on a multicenter randomized controlled trial for comprehensive interventions in 30 primary schools in China to evaluate the effects on blood pressure, which lasted for two semesters. The standards used for the diagnosis of high blood pressure were the cut-off points based on age and sex for Chinese children. Compared with the control group, the intervention effects were −0.5 mm Hg (95% confidence interval (CI): −1.1, 0; *p* = 0.064) for diastolic blood pressure and −0.9 mmHg (95% CI: −1.5, −0.3; *p* = 0.005) for systolic blood pressure. For the incidence of high blood pressure, the changes were −1.4% in the intervention group and 0.4% in the control group (1.8% difference between the two groups, *p* = 0.015) after trial. The school-based comprehensive interventions appeared to have moderate effects on high blood pressure prevention among children in China.

## 1. Introduction

With childhood hypertension increasingly becoming a public health problem globally [1], the prevalence of elevated blood pressure (BP) among children in China is also showing an increasing trend [2]. The elevated BP in childhood is associated with not only a high risk of hypertension [3,4] but also a high risk of coronary heart disease and premature mortality in adulthood [5]. In addition to gender, age, body shape, family history of hypertension, changes in dietary habits and stress [6,7], obesity is also a very important risk factor for elevated BP during childhood. Several studies have revealed a strong association between elevated BP and body mass index (BMI) [8], and the prevalence of elevated BP increases progressively with increasing BMI.

Nutrition education and physical activity are two important obesity intervention techniques among children. Previous studies revealed that community-based lifestyle intervention (nutrition and healthy lifestyles education) could ameliorate the usual blood pressure increase with age in children and young adults in the general population significantly [9], but short-term exercise intervention (<8 weeks) did not appear to reduce systolic and diastolic blood pressure in children [10]. Burke et al., 1995 found that nutrition education combined with physical activity could be the most successful approach to modifying lifestyle in children [11]. The CHILDREN study revealed that school-based dietary behaviors and physical activity intervention could reduce blood pressure by changing the fruit and fat/oil intake [12], and another school-based exercise intervention in Germany also showed the beneficial effects on blood pressure after a 37-week experimental period in children [13].

Few studies have verified the effect of interventions focused on obesity prevention by combining nutrition education and physical activity on blood pressure among children in developing countries. One multicenter randomized controlled trial of a school-based comprehensive intervention including nutrition education and physical activity focusing on childhood obesity prevention was implemented in China [14]. A moderately significant effect on the prevalence of overweight combined with obesity was observed after the intervention. We hypothesized that the comprehensive interventions would also cause a beneficial reduction in blood pressure levels among children in this cluster-randomized trial. Thus, this study aimed to analyze the effect of comprehensive interventions on blood pressure (mm Hg) and the relationship between changes in BMI and BP among Chinese primary school children. In order to help explain the effect of comprehensive intervention on dietary behaviors, food consumption was analyzed as explanatory variables in this study.

## 2. Materials and Methods

### 2.1. Study Design

This study was a school-based multicenter cluster randomized control trial. Five centers, including Shanghai, Chongqing, Guangzhou, Jinan and Harbin, were recruited. A two-step cluster sampling method was used for participant selection. First, six schools from each city were selected and randomly sorted into 2 groups (3 for control and 3 for intervention) in a lottery. In total, 30 primary schools were included in this trial. Second, two classes were selected from each grade (1st to 5th) in every sample school. All the schools selected into this program were public and located in metropolis; most of the families were above the average socio-economic level in China. This program lasted for 2 semesters from May 2009 to May 2010. No intervention was conducted in the control schools. The detailed design information about this trial has been reported in previous articles [14,15].

The inclusion criteria for selecting schools were as follows: (1) non-boarding school; (2) providing school lunch service, with more than 50% of students having lunch at school; and (3) the prevalence of obesity above 10% (being obtained from the annual routine physical examination for students before the trial). The exclusion criteria for participants were as follows: (1) students who participated or planned to participate in other similar intervention programs in the past year or the next year, respectively; (2) students who suffered from serious illnesses (such as congenital heart disease or joint replacement surgery) or who could not withstand severe physical activity or diet control.

This trial was approved by the Ethical Review Committee of the National Institute for Nutrition and Food Safety, Chinese Centre for Disease Control and Prevention. Informed consent was voluntarily signed by the participants’ parents or their guardians. The trial was registered in the Chinese Clinical Trial Register (number ChiCTR-PRC-09000402).

**Figure 1:**
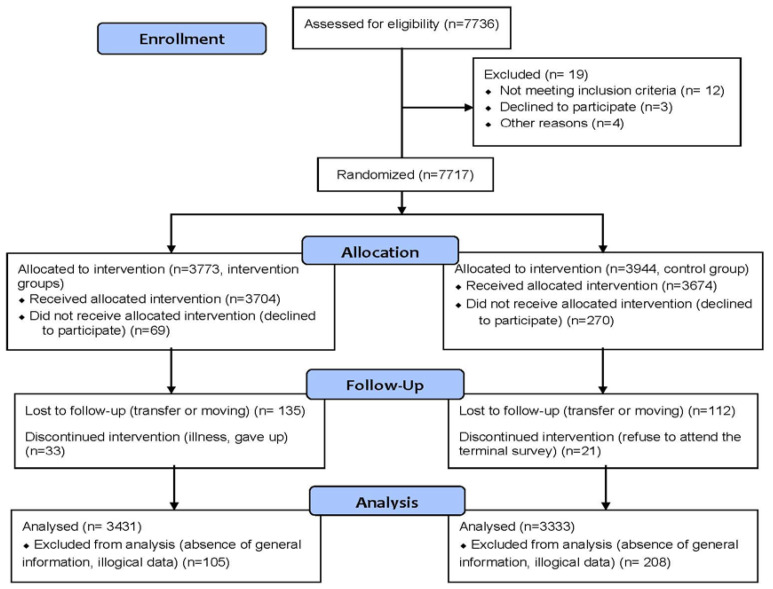
The CONSORT flowchart of the study.

The current analysis of the effect on high blood pressure was not the primary outcome of this trial. The calculation of sample size was performed according to the effected changes in blood pressure for 1.5 mm Hg in the intervention group [9]. The interclass correlation coefficient was 0.18. With a sample size of 5644, we would have 90% power to detect an effect size of as much as 0.7 from 30 schools located in 5 centers at both sides of the 0.05 level. Our effect size was 0.95 with a sample size of 6764, which was stronger than the minimum detectable level. The trial profile for the data analysis is shown in Figure 1.

### 2.2. Intervention Measures

Both nutrition education and physical activity interventions were included in the comprehensive interventions. Detailed information on the intervention measures can be found in previous articles [14,15]. A nutrition education handbook was developed for the nutrition education intervention [16]. Carton pamphlets were distributed to each student in the intervention schools. Nutrition and health courses were given 6 times for students, 2 times for parents and 4 times for teachers and health workers. The menu of school lunch for students in the school cafeteria was evaluated periodically, and specific nutrition improvements were suggested accordingly. Nutrition education was also targeted to parents, teachers and health workers in the intervention schools. A classroom-based physical activity program for primary students named “Happy 10” was used for physical activity intervention [17]. The students performed “Happy 10” with 10-min segment, moderate intensity, age- and space-appropriate exercises in school. The activities included games, dance and rhythmic gymnastics. Students were also encouraged to develop more forms of exercise that they liked. Furthermore, education about physical activity was provided to the students, parents, health workers and teachers. Students practiced “Happy 10” twice a day or 20 min once each school day.

Staff from the National Institute for Nutrition and Food Safety trained the group members from five centers for five days. Then, the members trained teachers and/or health workers for two days. They learned how to integrate the program into the school curriculum, and how to perform the activities. Slides and videos about the interventions were prepared by the program Office and provided to school teachers. Teachers revised the lessons to ensure that they understood the recommended techniques and strategies for implementation. The “Happy 10” program has been implemented and promoted in urban Beijing since 2004 and is a useful strategy for increasing physical activity among school children. The parents were involved in our study to improve the home environment. After obtaining permission, we applied the supervising strategies to make sure the interventions would be fully implemented by frequent visiting without notice to intervention schools. There were also some competing activities and seminars held between centers and schools.

### 2.3. BP and Other Anthropometric Measurements

A physical examination was conducted in the schools. BP was measured in the seated position using a mercury sphygmomanometer by trained nurses with at least a 10-min rest period before the measurement. The first and fifth Korotkoff sounds were used to represent systolic blood pressure (SBP) and diastolic blood pressure (DBP), respectively. Three measurements were taken for all participants with 2-min intervals, and the average of the two nearest measurements (≤2 mmHg) was used for the analysis. The standards being used for the diagnosis of high BP were the cut-off points based on age and sex for Chinese children and adolescents [18]. High BP was defined as DBP or SBP ≥ P_95_ cut-off points. Height was measured to an accuracy of 0.1 cm with a free-standing stadiometer mounted on a rigid tripod (GMCS-I, Xindong Huateng Sports Equipment Co., Ltd., Beijing, China). One overnight fasting body weight was measured to the nearest 0.1 kg on a digital scale (RGT-140, Wujin Hengqi Co., Ltd., Changzhou, China). Body mass index (BMI) was calculated as weight in kilograms divided by height in meters squared (kg/m^2^). Overweight and obesity were defined according to the China national standard for screening for overweight and obesity among school-age children and adolescents with BMI at baseline [19]. The energy expenditure was measured with energy monitor (UX-02, Beijing Yaohua Technology Development Co., Ltd., Beijing, China). Limited by the number of energy monitors and wearing requirements, only some of the students wore the energy monitors.

### 2.4. Sociodemographic Information and Food Consumption

Sociodemographic information was collected with a parent questionnaire. Parental education level was defined by maternal education level when available, which was supplemented by paternal education level as a surrogate when maternal education level was not available. The parent educational level was categorized into 3 levels: low level (illiterate), middle level (primary or junior middle school) and high level (senior middle school or above). The family’s income level was classified as household income per capita monthly in 2009. Food consumption information was collected with a 24-h dietary recall method for three consecutive days (two weekdays and one weekend day), and the records were administered by the subjects themselves or with their parents’ help.

### 2.5. Statistical Methods

The primary and second outcomes in this study were the changes in the BP mm Hg, and the incidence of high BP, respectively. The continuous variables were expressed as the mean and standard deviation. A mixed model (MM) was used for comparison of the means and the changes in continuous variables between groups. The repeated-measure method was used for comparison of the within-group means. Considering that the high BP, obesity(overweight), sex and age may affect the intervention effect, the analyzation of intervention effect in different subgroups was carried out. The interaction effect was analyzed with the model MM for BP mm Hg and generalized linear mixed model (GLMM) for the incidence of high BP. The incidence of high BP was analyzed using the GLMM by intention to treat. The MM and GLMM were used for the comparison of energy expenditure and the proportion of food consumption between groups, respectively. A *t*-test and chi-square test were used for the comparison of continuous variables and categorized variables between groups, respectively. Parental education level and family income level were adjusted for comparing between groups. Pearson correlation and linear regression models were used for the relationship between BMI changes and BP changes. The regression coefficients were estimated using Equation (1):

(1) $$y_{i}=\beta_{0}+\beta_{1}x_{i}+\beta_{n}x_{ni}\;,$$

In Equation (1), *y_i_* is the change in BP from baseline to end for child *i*; *β*_0_ is the intercept parameter; *x_i_* is the change in BMI from baseline to end for child *i*; *β*_1_ is the regression coefficient between BMI changes and BP changes; and *x_ni_* is the adjusted variable. The statistical significance level was set at *p* < 0.05. SAS software package version 9.4 (SAS Institute Inc., Cary, NC, USA) was used for analysis.

## 3. Results

### 3.1. General Characteristics

In the original program, 7717 children (3803 girls) were enrolled at baseline. Of these children, 6764 children (3398 girls) who completed the trial were used for the analyzation in this study. The average age was 9.1 (1.4) years old at baseline for students in both the control and intervention groups, and was 7.2 (0.4), 8.2 (0.4), 9.2 (0.4), 10.2 (0.4) and 11.2 (0.4) years old for students in 1st to 5th grade, respectively. There was no significant difference in SBP or DBP or the incidence of high SBP, high DBP or high BP between the control and intervention groups at baseline. Table 1 shows the characteristics of the participants, including age, sex, parental educational level and family income level. Though 906 participants were not reanalyzed because of being lost or missing information at the endpoint of the trial, no significant differences for the proportions of high BP and overweight/obesity were found between the lost and retained participants according to the baseline comparisons.

### 3.2. Energy Expenditure and Food Consumption

The energy expenditure data were collected from 605 participants (301 for control and 304 for intervention). After intervention, the energy expenditure increased by 63.5 kcal/day and 77.0 kcal/day in control and intervention groups, respectively (*p* = 0.967). The changes of food consumption are shown in Table 2. The proportion of participants with fruits consumption ≥1 time/3 days decreased by 4.0% in the control group but increased by 4.9% in the intervention group (*p* < 0.001). For the cereal, meat, fish and dried legume consumption, the proportion changes were −12.5 and −6.1% (>3 times/3 days, *p* = 0.005), −0.9 and 4.4% (>3 times/3 days, *p* = 0.018), −3.4 and −9.2% (>1 times/3 days, *p* = 0.013) and 3.0 and −11.5% (>1 times/3 days, *p* < 0.001) in the control and intervention groups, respectively.

### 3.3. Effects on BP

The effects on DBP and SBP are shown in Table 3. Both DBP and SBP increased significantly after one school year within groups for the intervention and control group. The analysis of the effect on BP indicated that the intervention effect was significant for DBP in the baseline high BP subgroup (−2.4 mm Hg; 95% CI: −4.3, −0.6; *p* = 0.010), baseline overweight/obesity subgroup (−1.9 mm Hg; 95% CI: −3.1, −0.7; *p* = 0.002) and younger children subgroup (−0.7 mm Hg; 95% CI: −1.3, −0.2; *p* = 0.012), and for SBP in the baseline non-high BP subgroup (−1.1 mm Hg; 95% CI: −1.7, −0.5; *p* = 0.001), baseline overweight/obesity subgroup (−1.5 mm Hg; 95% CI: −2.8, −0.2; *p* = 0.026), girls subgroup (−1.0 mm Hg; 95% CI: −1.9, −0.2; *p* = 0.017) and younger children subgroup (−0.8 mm Hg; 95% CI: −1.4, −0.1; *p* = 0.016).

Table 4 shows the intervention effects on the incidence of high BP. The incidence of high BP decreased by 1.4% from 10.0% in the intervention group and increased by 0.4% from 10.9% in the control group, compared with the control group, with a 1.8% decrease in the intervention group, *p* = 0.015. The effect was still significant in the overweight/obesity (at baseline) and female subgroups. The changes in high BP incidence in the intervention and control groups were −3.7 and 2.0 for overweight/obese (5.7% difference between groups, *p* = 0.009) and −2.7 and 0.3 for girls (3.0% difference between groups, *p* = 0.007), respectively.

### 3.4. Relationship between BMI and BP

**Figure 2:**
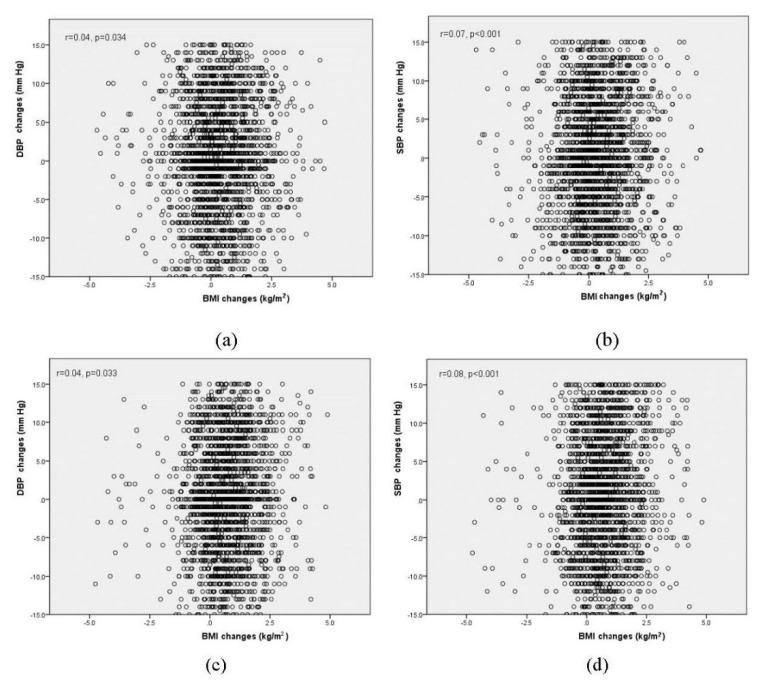
The scatterplots for BMI changes and BP changes in intervention and control groups: (**a**) BMI and DBP in intervention group, (**b**) BMI and SBP in intervention group, (**c**) BMI and DBP in control group, (**d**) BMI and SBP in control group.

The correlation relationship between BMI changes and BP changes is shown in Figure 2. A significant positive correlation between them was found, with coefficients of 0.04 (*p* = 0.034) and 0.04 (*p* = 0.033) between BMI and DBP in the intervention and control groups, respectively, and 0.07 (*p* < 0.001) and 0.08 (*p* < 0.001) between BMI and SBP in the intervention and control groups, respectively.

The regression coefficients between BMI changes and BP changes are shown in Table 5. With increasing BMI, a significant incline effect was found on DBP with coefficients of 0.24 (*p* = 0.034) and 0.28 (*p* = 0.033) in the intervention and control groups and on SBP with coefficients of 0.47 (*p* < 0.001) and 0.72 (*p* < 0.001) in the intervention and control groups, respectively. Table 6 presents the sensitivity analysis results. After adjusting for baseline BMI, baseline BP and both of them in different models, the coefficient was still significant and weaker in the intervention group model than in the control group model.

## 4. Discussion

The results indicated that the school-based comprehensive interventions including nutrition education and physical activity had a mild beneficial effect on high blood pressure among children in this study. After one school year, the comprehensive interventions resulted in a 1.4% decrease in high blood pressure incidence in the intervention group but a 0.4% increase in the control group. Although this represented a modest shift in the mean blood pressure of the children, the intervention significantly decreased the incidence rate of high BP. We believe that this intervention has a positive primary prevention effect and significant public policy implications.

A previous study indicated that 60 min exercise three times per week over three months could lead to an average 7% decrease in systolic blood pressure and a 12% decrease in the rate of hypertension among prepubertal obese children [20]. Another community-based lifestyle intervention for BP in children and young adults in the general population of Pakistan for two years resulted in BP 1.6/1.4 mm Hg lower in the intervention group than in the control group [9]. In the USA, the CATCH program modified the fat content of school lunches, increased moderate-to-vigorous physical activity and improved eating and physical activity behaviors in children over three school years. However, BP did not show any beneficial changes: the systolic/diastolic BP increased by 4.8/2.4 mm Hg and 4.8/2.1 mm Hg in the intervention and control groups, respectively [21]. Another school-based intervention study including exercise and education in the USA indicated that the systolic and diastolic blood pressures increased more in the control group than in the intervention group [22]. In Greece, the CHILDREN study focused on obesity and BP among primary school students: by increasing the physical activity, availability of fruits and vegetables and parental support, both systolic and diastolic BP decreased in the intervention group (−1.6/−0.5 mm Hg) but increased in the control group (1.9/2.3 mm Hg) after 12-month intervention [12]. However, the results did not show a significant decrease in either DBP or SBP totally in our study, but a smaller increase for SBP was found in the intervention group compared to the control group which explained the favorable intervention effect, which may be caused by two factors—the relatively short duration of the intervention (one school year) and the relatively modest amount of physical activity (20 min each school day), even though a significant intervention effect of 46.0 min/week for self-reported physical activity time has been reported previously [14]. The physical activity time increased from 815.9 ± 420.6 min/week to 855.1 ± 405.6 min/week in the intervention group, and energy expenditure increased 14.5 kcal more than the control group every day [14], which coupled with the modification in dietary behavior caused the beneficial intervention effect on BP. Dietary interventions could reduce and control the incidence of hypertension mainly by reducing sodium intake [23,24]. The association between excessive dietary sodium and hypertension has been extensively investigated. Children with hypertension should be encouraged to eat a diet high in fresh fruits and vegetables, fiber and low-fat dairy in addition to a reduction in sodium intake [25,26]. The intervention provided nutritional knowledge to the students, and their parents and the intervention had a favorable effect on the proportion of children who ate fruits and cereals regularly, which could have contributed to the effect seen on high blood pressure prevention in our study.

Some studies have demonstrated that increasing BMI, obesity and abdominal circumference are correlated with increased hypertension rates in children [27], which is consistent with our findings. Though the correlation between the increasing BMI and the increasing BP was significant, it was very weak in this study. We also found that the correlation was stronger in the control group than in the intervention group. The correlation strength may have been affected by comprehensive intervention, and the sensitivity of BP increasing with increased BMI decreased after intervention. Children with high BP should make their lifestyle change to lower BP and reduce the risk of developing additional cardiovascular disease risk factors [20,28,29,30]. For overweight or obese children, weight loss should be encouraged by comprehensive intervention programs. Various studies have suggested that with an intervention duration of three to six months, it is possible to generate effectiveness [25]. Though one school year ensured the necessary duration in our study, we still consider that it is not long enough to compare with other effective programs.

There were some limitations in our study. First, the results were not the primary outcome of the comprehensive intervention trial; the trial was originally designed for childhood obesity prevention. However, the analysis was still valid based on the calculation of the effect size in our methods section. Second, the covered children were not followed up after the program to assess the long-term impact on their lifestyle, so we do not know how persistent it is. Tracking the children is very difficult in practice: students would attend different middle schools after graduating from primary schools. This will bring great challenges to follow-up. Third, the detailed energy and nutrient intake was not analyzed for the students—only the frequency of different food consumption was analyzed before and after intervention. Another limitation was that the intervention covered only urban children and not rural children. The prevalence of childhood obesity and hypertension has also been increasing more quickly in rural areas [31,32,33]—it is uncertain whether this intervention model was suitable for rural children. In particular, Chinese rural children have been experiencing a nutrition transition in recent years [34].

## 5. Conclusions

In conclusion, we observed significantly moderate intervention effects on the prevention of high BP among children. A school-based comprehensive intervention approach that involves implementing school policies and practices that improve both nutrition knowledge and physical activity level should be encouraged in China for both the prevention of childhood obesity and prevention of high BP among school children.

## Figures and Tables

**Table 1:** Characteristics of the participants at baseline.

Characteristics	Intervention or Control		Retained or Lost	
	Intervention Group	Control Group	Retained	Lost
No. of participants	3431	3333	6764	906
Sex, girls ^#^ (*n* (%))	1693 (49.3)	1648 (49.4)	3341 (49.4)	438 (48.3)
Overweight/obese ^#^ (*n* (%))	905 (26.4)	826 (24.8)	1731 (25.6)	223 (24.6)
DBP ^†^ (mm Hg, (Mean (SD))	62.5 (9.3)	62.4 (9.1)	62.5 (9.2)	61.1 (9.0)
SBP ^†^ (mm Hg, (Mean (SD))	99.2 (11.0)	99.0 (11.3)	99.1 (11.1)	97.1 (11.3)
High BP ^#^ (*n* (%))	360 (10.5)	368 (11.0)	728 (10.8)	73 (8.1)
Age ^†^ (years, (Mean (SD))	9.1 (1.4)	9.1 (1.4)	9.1 (1.4)	9.1 (1.4)
Children ≤ 10 years old ^#^ (*n* (%))	3092 (90.1)	3016 (90.5)	6108 (90.3)	802 (88.5)
Parental educational level ^#^ (*n* (%))				
Low (illiterate)	6 (0.2)	11 (0.4) **	17 (0.3)	2 (0.3) *
Middle (primary or junior middle school)	889 (33.3)	1020 (39.8)	1909 (36.4)	191 (33.2)
High (senior middle school or above)	1777 (66.5)	1530 (59.7)	3307 (63.3)	382 (66.4)
Family’s income level (yuan/month/per capita) ^#^ (*n* (%))				
≤1500	1094 (41.0)	1160 (45.3) **	2254 (43.1)	235 (40.9) *
1501–2500	756 (28.4)	683 (26.7)	1439 (27.7)	154 (26.8)
>2500	816 (30.6)	715 (28.0)	1531 (29.1)	186 (32.3)

BP, blood pressure. ** *p* < 0.01. * *p* < 0.05.^#^ Comparison by chi-square test. ^†^ Comparison by *t* test.

**Table 2:** The energy expenditure and different food consumption among participants.

Variables	Control Group		Intervention Group		*p*-Value
	Baseline	Change	Baseline	Change	
Energy expenditure (Kcal/day, mean (SD))	236 (128.9)	63.5 (129.9)	224.9 (131.4)	77.0 (149.1)	0.967
Cereals (>3 times/3 days, *n* (%))	1324 (80.8)	−205 (−12.5)	1337 (78.1)	−105 (−6.1)	0.005
Meat (>3 times/3 days, *n* (%))	1001 (61.1)	−14 (−0.9)	1098 (64.1)	75 (4.4)	0.018
Vegetables (>3 times/3 days, *n* (%))	1099 (67.1)	−15 (−1.0)	1168 (68.2)	−20 (−1.1)	0.883
Fruits (≥1 time/3 days, *n* (%))	765 (46.7) *	−65 (−4.0)	741 (43.3)	84 (4.9)	0.000
Dairy (≥1 time/3 days, *n* (%))	1124 (68.6)	−94 (−5.8)	1217 (71.1)	−85 (−5.0)	0.705
Eggs (≥1 time/3 days, *n* (%))	1223 (74.6)	59 (3.6)	1314 (76.8)	11 (0.6)	0.169
Fish and shellfish (≥1 time/3 days, *n* (%))	927 (56.6) *	−55 (−3.4)	1041 (60.8)	−157 (−9.2)	0.013
Fungi and algae (≥1 time/3 days, *n* (%))	600 (36.6)	−15 (−0.9)	603 (35.2)	−10 (−0.6)	0.850
Dried legumes (≥1 time/3 days, *n* (%))	874 (53.3) **	48 (3.0)	1018 (59.5)	−197 (−11.5)	<0.001
Nuts and seeds (≥1 time/3 days, *n* (%))	284 (17.3)	−61 (−3.7)	215 (12.6)	−57 (−3.4)	0.514
Snack (≥1 time/3 days, *n* (%))	305 (18.6)	−39 (−2.4)	313 (18.3)	−48 (−2.8)	0.764
Fast foods (≥1 time/3 days, *n* (%))	1145 (69.9)	−113 (−6.9)	1178 (68.8)	−97 (−5.7)	0.579
Beverages (≥1 time/3 days, *n* (%))	586 (35.8) **	−184 (−11.3)	527 (30.8)	−156 (−9.1)	0.420
Sugars and preserves (≥1 time/3 days, *n* (%))	404 (24.6) **	−64 (−3.9)	353 (20.6)	−33 (−1.9)	0.669

The mixed model and generalized linear mixed model were used for the comparison of energy expenditure and food consumption between groups after economical level and parent educational level were adjusted, respectively. * *p* < 0.05 and ** *p* < 0.01 for the comparison between control and intervention groups at baseline.

**Table 3:** The intervention effect on BP (mm Hg).

	Intervention Group		Control Group		Intervention Effect	
Groups	Baseline (Mean ± SD)	Change (Mean ± SD)	Baseline (Mean ± SD)	Change (Mean ± SD)	Beta (95%CI)	*p*-Value
DBP						
Total	62.5 ± 9.3	1.3 ± 11.7 **	62.4 ± 9.1	1.8 ± 11.4 **	−0.5 (−1.1, 0)	0.064
High BP at baseline	77.5 ± 8.3	−10.6 ± 12.6 **	76.4 ± 8.4	−8.1 ± 12.8 **	−2.4 (−4.3, −0.6)	0.010
Non-high BP at baseline	60.8 ± 7.7	2.7 ± 10.8 **	60.7 ± 7.6	3.0 ± 10.6 **	−0.4 (−0.9, 0.2)	0.186
*p*-value for interaction					<0.001	
Overweight/obese at baseline	65.8 ± 9.9	0.3 ± 12.7	65.1 ± 9.9	2.2 ± 12.2 **	−1.9 (−3.1, −0.7)	0.002
Non-overweight/obese at baseline	61.4 ± 8.9	1.6 ± 11.4 **	61.5 ± 8.7	1.7 ± 11.2 **	0 (−0.7, 0.6)	0.903
*p*-value for interaction					<0.001	
Boys	62.5 ± 9.0	1.9 ± 11.4 **	62.2 ± 9.0	2.4 ± 11.1 **	−0.4 (−1.2, 0.3)	0.275
Girls	62.6 ± 9.7	0.6 ± 12.1 *	62.6 ± 9.3	1.2 ± 11.8 **	−0.6 (−1.4, 0.2)	0.127
*p*-value for interaction					<0.001	
Children ≤ 10 years	62.3 ± 9.3	1.0 ± 11.7 **	62.0 ± 9.0	1.8 ± 11.4 **	−0.7 (−1.3, −0.2)	0.012
Children > 10 years	64.4 ± 9.5	3.7 ± 11.8 **	66.4 ± 9.4	2.2 ± 12.0 **	1.4 (−0.4, 3.3)	0.123
*p*-value for interaction					<0.001	
SBP						
Total	99.2 ± 11.0	1.9 ± 12.2 **	99.0 ± 11.3	2.7 ± 13.0 **	−0.9 (−1.5, −0.3)	0.005
High BP at baseline	116.6 ± 8.8	−8.6 ± 12 **	117.1 ± 9.6	−8.7 ± 12.8 **	0.1 (−1.7, 2.0)	0.874
Non-high BP at baseline	97.1 ± 9.3	3.1 ± 11.6 **	96.7 ± 9.2	4.1 ± 12.2 **	−1.1 (−1.7, −0.5)	0.001
*p*-value for interaction					<0.001	
Overweight/obese at baseline	104.4 ± 11.3	1.9 ± 13.1 **	104.4 ± 12.6	3.4 ± 14.4 **	−1.5 (−2.8, −0.2)	0.026
Non-overweight/obese at baseline	97.3 ± 10.2	1.9 ± 11.8 **	97.2 ± 10.2	2.5 ± 12.4 **	−0.7 (−1.3, 0)	0.056
*p*-value for interaction					<0.001	
Boys	100.2 ± 10.9	2.0 ± 12.2 **	100.2 ± 11.3	2.7 ± 12.8 **	−0.7 (−1.5, 0.2)	0.118
Girls	98.1 ± 11.0	1.7 ± 12.2 **	97.7 ± 11.1	2.8 ± 13.1 **	−1.0 (−1.9, −0.2)	0.017
*p*-value for interaction					<0.001	
Children ≤ 10 years	98.9 ± 10.9	1.6 ± 12 **	98.6 ± 11.2	2.4 ± 12.8 **	−0.8 (−1.4, −0.1)	0.016
Children > 10 years	101.6 ± 11.1	4.3 ± 13.6 **	102.7 ± 11.5	6.1 ± 13.7 **	−1.9 (−3.9, 0.2)	0.083
*p*-value for interaction					<0.001	

The mixed model was used for comparison between groups. The repeated-measure method was used for comparison of the within-group means. * *p* < 0.05, ** *p* < 0.01. High BP, DBP or SBP ≥ P_95_ cut-off points.

**Table 4:** The intervention effect on the incidence of high BP.

Groups	Intervention Group		Control Group		Intervention Effect	
	Baseline (*n* (%))	Change (*n* (%))	Baseline (*n* (%))	Change (*n* (%))	Beta (95%CI)	*p*-Value
Total	392 (10.0)	−55 (−1.4)	409 (10.9)	17 (0.4) *	−0.2 (−0.5, 0)	0.015
Non-high BP at baseline	0 (0)	251 (7.1) **	0 (0)	321 (9.6) **	−0.3 (−0.5, −0.1)	<0.001
High BP at baseline	392 (100)	−306 (−78.1) **	409 (10.9)	−304 (−74.3) **	−0.2 (−0.5, 0.1)	0.207
*p*-value for interaction					0.576	
Non-overweight/obese	180 (6.3)	−16 (−0.6)	205 (7.2)	−1 (0)	−0.1 (−0.4, 0.1)	0.347
Overweight/obese	212 (20.4)	−39 (−3.7)	204 (22.3)	18 (2.0) *	−0.4 (−0.7, −0.1)	0.009
*p*-value for interaction					<0.001	
Boys	178 (9)	−2 (−0.1)	193 (10.1)	12 (0.7)	−0.1 (−0.4, 0.2)	0.470
Girls	214 (11.1)	−53 (−2.7) *	216 (11.6)	5 (0.3)	−0.4 (−0.7, −0.1)	0.007
*p*-value for interaction					0.021	
Children ≤ 10 years	358 (10.2)	−50 (−1.4)	372 (11.0)	2 (0)	−0.2 (−0.4, 0)	0.055
Children > 10 years	34 (8.7)	−5 (−1.3)	37 (10.1)	15 (4.1) **	−0.6 (−1.3, 0.1)	0.076
*p*-value for interaction					0.003	

The intention to treat analyzation was used. The generalized linear mixed model was used for the comparison between groups, and within groups. * *p* < 0.05, ** *p* < 0.01. High BP, DBP or SBP ≥ P_95_ cut-off points.

**Table 5:** The regression efficiency (*β*_1_) between body mass index (BMI) changes and BP changes.

Variables	Intervention Group		Control Group	
	*β*_1_	*p*-Value	*β*_1_	*p*-Value
DBP-C	0.24	0.034	0.28	0.033
SBP-C	0.47	<0.001	0.72	<0.001

BMI, body mass index. BP, blood pressure. DBP-C = diastolic blood pressure at the end—diastolic blood pressure at baseline. SBP-C = systolic blood pressure at the end—systolic blood pressure at baseline.

**Table 6:** The sensitivity analysis for regression efficiency (*β*_1_) between BMI changes and BP changes.

Models	Intervention Group		Control Group	
	*β*_1_	*p*-Value	*β*_1_	*p*-Value
DBP-C				
Model 1	0.22	0.053	0.27	0.041
Model 2	0.48	<0.001	0.75	<0.001
Model 3	0.67	<0.001	0.68	<0.001
SBP-C				
Model 4	0.50	<0.001	0.70	<0.001
Model 5	0.71	<0.001	1.29	<0.001
Model 6	1.05	<0.001	1.22	<0.001

BP, blood pressure. DBP-C = diastolic blood pressure at the end—diastolic blood pressure at baseline. SBP-C = systolic blood pressure at the end—systolic blood pressure at baseline. Model 1 adjusted for BMI at baseline. Model 2 adjusted for DBP at baseline. Model 3 adjusted for both DBP and BMI at baseline. Model 4 adjusted for BMI at baseline. Model 5 adjusted for SBP at baseline. Model 6 adjusted for both SBP and BMI at baseline.
